# TMSB10 drives prostate cancer aggressiveness via immune microenvironment regulation

**DOI:** 10.1186/s10020-025-01211-8

**Published:** 2025-04-30

**Authors:** Haoran Xia, Jiaxin Ning, Xiaoxiao Guo, Hongchen Song, Xuanhao Li, Xuan Wang

**Affiliations:** 1 Department of Urology, Beijing Friendship hospital, Capital Medical University, Beijing, P. R. China; 2 Institute of Urology, Beijing Municipal Health Commission, Beijing, P. R. China; 3 Department of Urology, Beijing Hospital, National Center of Gerontology, Institute of Geriatric Medicine, Chinese Academy of Medical Sciences, Beijing, P. R. China

**Keywords:** TMSB10, Prostate cancer, Tumor immune microenvironment, Macrophage polarization, Immune evasion, Cancer therapy

## Abstract

Thymosin β10 (TMSB10) has emerged as a key player in the progression of prostate cancer, significantly influencing the tumor immune microenvironment. Pan-cancer analysis from The Cancer Genome Atlas (TCGA) revealed that TMSB10 is upregulated across multiple cancer types, particularly in prostate cancer, where high TMSB10 expression correlates with poorer patient outcomes. Functional assays using prostate cancer cell lines LNCaP and DU145 showed that TMSB10 silencing suppresses cell proliferation, migration, and invasion, while overexpression enhances these oncogenic processes. Furthermore, co-culture experiments demonstrated that TMSB10 overexpression skews macrophage polarization, decreasing the population of M1-type macrophages while increasing M2-type macrophages. This shift reduces immune cell cytotoxicity and alters cytokine secretion, highlighting TMSB10’s role in immune evasion. These findings establish TMSB10 as a pivotal factor in prostate cancer biology, promoting tumor aggressiveness and modulating the immune response within the tumor microenvironment. TMSB10 presents a promising therapeutic target for prostate cancer, offering new avenues for treatments aimed at altering the tumor immune landscape. This research also provides a foundation for further exploration of TMSB10’s role in other cancers.

**Graphical abstract:**

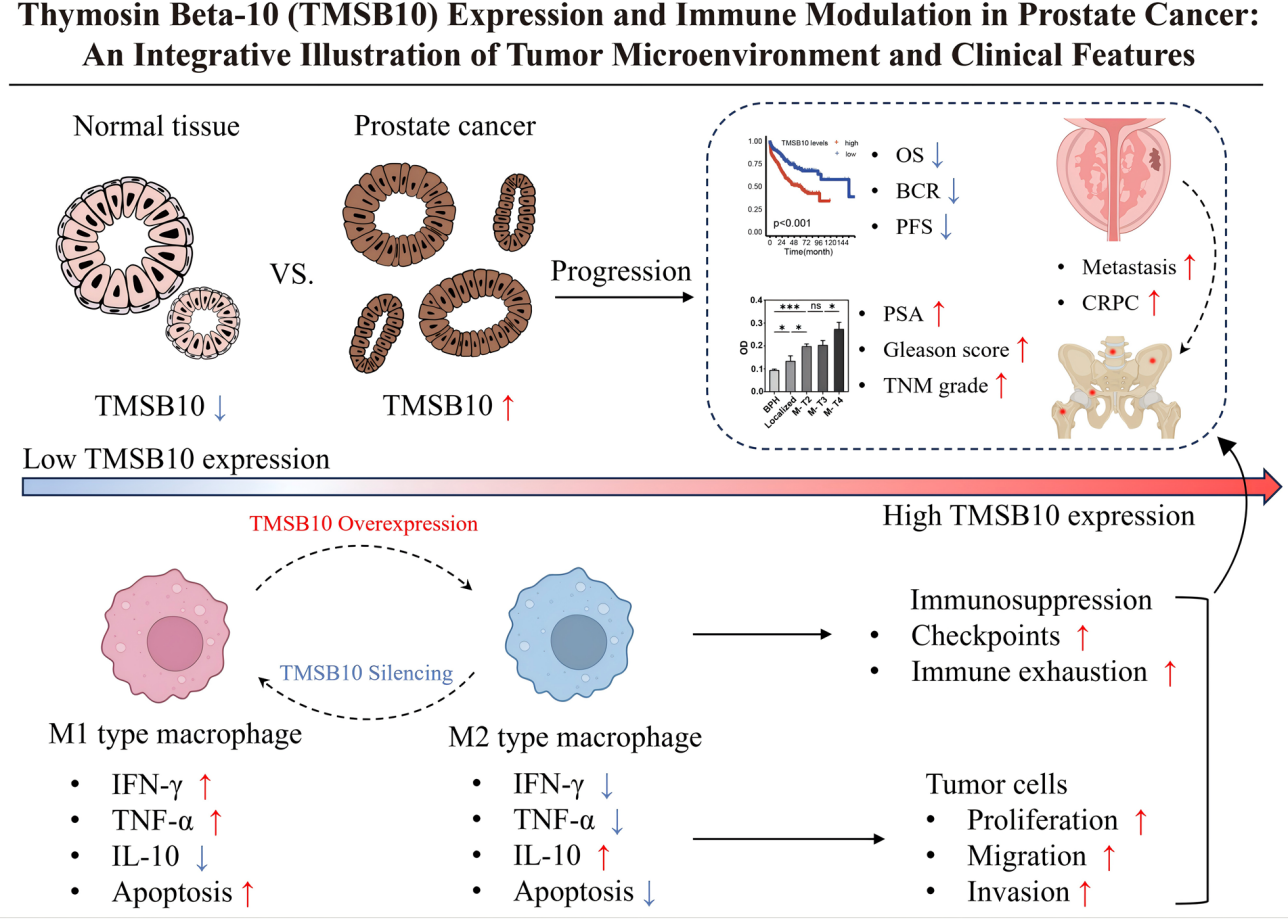

**Supplementary Information:**

The online version contains supplementary material available at 10.1186/s10020-025-01211-8.

## Introduction

Prostate cancer, as one of the principal types of male malignancies globally, has garnered widespread attention due to its rising incidence and mortality rates (Sung et al. 2021; Sekhoacha et al. 2022; Bergengren et al. 2023). According to statistics, in the past 2023, new cases of prostate cancer in the United States accounted for 29% of male cancers, and the estimated number of deaths accounted for 11% of male cancer deaths (Schaeffer et al. 2023). Although the overall mortality rate has decreased over the past few decades, it has stabilized in recent years due to an increase in late-stage diagnoses. The diagnosis and treatment of prostate cancer pose numerous challenges: approximately 5% of patients are diagnosed with distant metastasis and are ineligible for curative surgery (Farolfi et al. 2021; Murthy et al. 2021; Tran et al. 2023). Currently, androgen deprivation therapy (ADT), radiotherapy, and chemotherapy are the primary treatment options for these patients (Le et al. 2023; Collins and Cheng 2023). However, most hormone-sensitive prostate cancers eventually progress to castration-resistant prostate cancer (CRPC) or develop resistance to radiotherapy and chemotherapy (Pernigoni et al. 2021; Yanagisawa et al. 2023; Fujita et al. 2022). In recent years, the link between immunotherapy and the treatment of prostate cancer, especially for patients who do not respond to ADT, radiotherapy, or chemotherapy, has become increasingly significant (Norz and Rausch 2021). However, it is essential to note that not all patients are sensitive to immunotherapy (Baker et al. 2023; Märkl et al. 2022; Lin et al. 2022). Therefore, there is an urgent need to identify new molecular biomarkers to improve prognosis prediction, tumor grading, progression, and treatment outcomes.

Thymosin β10 (TMSB10) is a crucial member of the β-thymosin family, primarily involved in multiple cellular biological processes such as cytoskeletal organization, morphology, proliferation, and motility (Pan et al. 2020). TMSB10 regulates actin polymerization and cell movement as an intracellular G-actin sequestering protein. Recent studies have found TMSB10 closely associated with hepatocyte differentiation and maturation and linked with immune cells and immune-related pathways (Li et al. 2022a, b; Liu et al. 2021). Notably, TMSB10’s expression in fetal testicular precursor cells, by inhibiting the Ras/Erk signaling pathway, promotes their transformation into fetal testicular cells, thus participating in androgen production. It suggests a close connection between TMSB10 and androgen-related diseases, such as prostate cancer. However, there is currently a lack of sufficient research on the role and mechanisms of TMSB10 in prostate cancer, which is particularly important in the study of biomarkers and therapeutic targets for this disease.

This study aims to explore the expression patterns, functions, and relationships of TMSB10 with tumor progression and immune regulation in prostate cancer. By integrating bioinformatics analysis with cytological and immunological techniques, this study will assess the expression levels of TMSB10 in prostate cancer and examine its correlation with patient prognosis. Through *in vitro* and *in vivo* experiments, this study will delve into the effects of TMSB10 on the proliferation, migration, and invasion of prostate cancer cells and its role in regulating the immune microenvironment. Special emphasis will be placed on determining whether TMSB10 expression influences immune modulation and if it could serve as a potential predictive marker for immunotherapy in prostate cancer. This study is the first to investigate the expression of TMSB10 in prostate cancer and its ability to predict poor prognosis, analyzing its biological functions, such as affecting the proliferation, migration, and invasion of LNCaP and DU145 cells, and its role in the tumor immune microenvironment state. These findings provide a new theoretical foundation for understanding the role of TMSB10 in the progression of prostate cancer and have significant scientific and clinical implications for the development of new therapeutic targets and for improving the clinical prognosis of prostate cancer patients.

## Materials and methods

### Data acquisition and preparation

We sourced transcriptome and gene mutation data matrices and clinical and prognostic information for 33 types of human cancers from The Cancer Genome Atlas (TCGA). This data was used to determine the differential expression of the TMSB10 gene across various tissues. Additionally, we calculated tumor mutational burden (TMB) and microsatellite instability (MSI) for each sample based on mutation information in the TCGA cohort. We also collected transcriptome data matrices and prognostic information from seven prostate cancer cohorts from the International Cancer Genome Consortium (ICGC) and the Gene Expression Omnibus, encompassing 1080 patients. Except for the GSE116918 cohort, which consisted of patients given radical radiotherapy/ADT, all other cohorts were comprised of patients who underwent prostatectomy. We utilized these cohorts to validate the role of the TMSB10 gene in the prognosis of prostate cancer.

Additionally, we included data from cohorts without prognostic information to analyze the differential expression of the TMSB10 gene. The downloaded transcriptome data were normalized to RNA-Seq data using the fragments per kilobase per million mapped reads (FPKM) standard. We conducted bioinformatics analysis using R software (version 4.1.2). Before further analysis, data was log2 transformed if necessary, and gene expression heterogeneity was corrected using surrogate variable analysis methods (R package “sva”).

### Survival analysis

We obtained normalized TMSB10 gene expression values and prognostic information and performed univariate Cox regression analysis in the TCGA cancer cohort to determine the prognostic value of TMSB10 across different prognostic indicators, including overall survival (OS), disease-free survival (DFS), and progression-free survival (PFS). Box plots were used to display differences in TMSB10 expression between tumor/normal tissues and across different grades/groups in 33 cancer types.

### Prognostic analysis

To further ascertain the prognostic value of TMSB10 in prostate cancer, patients in each cohort were divided into high and low-expression groups based on the median expression value of TMSB10. Kaplan-Meier curves, plotted using R packages “survival” and “SurvMiner,” illustrated the prognostic value of TMSB10 in OS, biochemical recurrence (BCR), and PFS. Patients from different cohorts with the same follow-up endpoints were combined. Kaplan-Meier curves were redrawn, followed by meta-analysis using the R package “meta” for more precise results.

### Clinical analysis

Initially, univariate and multivariate Cox regression analyses were conducted to study the independent prognostic value of TMSB10 among common clinical prognostic indicators associated with PFS and BCR. Subsequently, we collated clinical information from all prostate cancer cohorts and determined statistical differences between high and low TMSB10 expression groups. Based on the European Association of Urology (EAU) risk groups, patients in each cohort were classified into four groups: Group I: localized intermediate-risk, prostate-specific antigen (PSA) < 10 ng/mL, Gleason score (GS) < 7 (International Society of Urological Pathology (ISUP) grade 1) and cT1–2a; Group II: localized intermediate-risk, PSA 10–20 ng/mL, GS 7 (ISUP grades 2/3) or cT2b; Group III: localized high-risk, PSA > 20 ng/mL, GS > 7 (ISUP grades 4/5) or cT2c; Group IV: locally advanced, any PSA, any GS (any ISUP grade), and cT3–4 or cN+. Patients needing more information to determine their group were excluded from the analysis. To describe the differential expression of TMSB10 at different treatment stages of prostate cancer, we also analyzed cohorts including GSE178631, GSE169038, GSE134160, GSE21034, GSE77930, GSE150368, and GSE189343. Box plots (created using R packages “ggplot2,” “ggpubr,” and GraphPad Prism software) were used to observe statistical differences in TMSB10 gene expression between different groups.

### Immune infiltration and immunotherapy response

In the TCGA prostate cancer cohort, we employed various methods to assess the immune infiltration of samples. Cell typing and single-sample GSEA (ssGSEA) were initially conducted using immune gene sets obtained from molecular signature databases to evaluate immune infiltration. We further determined immune infiltration using immune cell abundance identifier (ImmuCellAI) and immune phenotype score (IPS). To illustrate the differences between TMSB10 expression groups, we used various charts, including heatmaps (with R package “pheatmap”), correlation plots, and lollipop charts (with R package “ggplot2”) to demonstrate differences in immune cell infiltration and immune-related gene expression. To further investigate immunotherapy’s impact on different TMSB10 expression groups, we downloaded IMvigor210 data, a publicly available immune therapy cohort for BLCA. We also extracted immune phenotypes of prostate cancer patients with different immune landscapes from the Cancer Immunome Atlas (TCIA) database. These data were used to analyze the immunotherapy response of different TMSB10 expression groups.

### Immunohistochemistry and immunofluorescence

Paraffin-embedded tissue samples from benign prostatic hyperplasia, localized prostate cancer, and prostate cancer bone metastases were processed for immunohistochemistry (IHC) and immunofluorescence (IF) to analyze TMSB10 and immune checkpoint expression. The supplementary materials provide detailed procedures for sample preparation, antibody incubation, and imaging analysis. The Research Ethics Committee of Beijing Hospital approved all tissue sample collection and research, with approval number 2022BJYYEC-350-01. All participants signed informed consent forms, allowing their samples to be used for this study.

### Cell culture and transfection

Prostate cancer cell lines (LNCaP, DU145, TRAMP-C1) and HEK-293 T cells were cultured under standard conditions. Gene manipulation was performed using transient transfection and a lentiviral overexpression system (detailed sequences, vectors, and transfection protocols are provided in Tables 1 and 2; additional information is available in the supplementary materials). Additionally, the lentiviral interference vector pSIH1-H1-copGFP (Cat# SI501 A-1, System Biosciences, USA) was used to construct a TMSB10 knockdown system, with lentiviral particles packaged using the lentivirus packaging kit (Cat# A35684 CN, Invitrogen, USA). Supernatants containing lentiviral particles were collected 48 hours post-transfection, with a final titer of 1×10^8^ TU/ml. The shRNA sequences used were as follows: sh-NC: AGGCTACAATGATCAGACTAAT; sh-TMSB10#1: AAACGGAGACGCAGGAGAA; sh-TMSB10#2: GAGAAGCGGAGUGAAAUUU; sh-TMSB10#3: CGACCAAAGAGACCAUUGA.

**Table 1:** Sequence of the small interfering RNA

TMSB10 siRNA	Sequence (5′–3′)
si-TMSB10#1	Sense: GAAAUCGCCAGCUUCGAUATT
	Anti-sense: UAUCGAAGCUGGCGAUUUCTT
si-TMSB10#2	Sense: CGACCAAAGAGACCAUUGATT
	Anti-sense: UCAAUGGUCUCUUUGGUCGTT
si-RNA-NC	Sense: UUCUCCGAACGUGUCACGUTT
	Anti-sense: ACGUGACACGUUCGGAGAATT

**Table 2:** TMSB10 overexpressed sequences

TMSB10-CDS sequence (5′–3′) NM_021103.4	
ATGGCAGACAAGCCGGACATGGGCGAAATCAACAGCTTCGATAAGGCCAAGCTGAAGAAGACGGAGACGCAGGAGAAGAACACCCTGCCCACCAAAGAGACCATTGAGCAGGAGAAGCAAGCCAAGTGA	

Sequence (https://www.ncbi.nlm.nih.gov/nuccore/NM_021103.4)

### Cell proliferation, migration, and apoptosis assays

Cell proliferation was assessed using the CCK-8 assay, while migration was evaluated with a Transwell system. Apoptosis and cell cycle analyses were conducted via flow cytometry, and Western blot was employed to examine related protein expression. Primer designs for RT-qPCR are provided in Table 3. Complete experimental procedures are described in the supplementary materials.

**Table 3:** The primer sequence of qRT-RCR

Target	Sequence (5′–3′)
TMSB10	F: TGGCAGACAAACCAGACATGGG
	R: TCACTCCGCTTCTCCTGCTCAA
CD274	F: GCCGAAGTCATCTGGACAAGC
	R: TGATTCTCAGTGTGCT-GGTCAC
PDL2	F: CCACACCGTGAAAGAGCCACTT
	R: CTGTCCTTCGTCCCT-CACTTGG
CD276	F: CTCCCTACTCGAAGCCCAGCAT
	R: TGCCAGAACACCTCA-GCCTCAG
IDO1	F: CTCACAGACCACAAGTCACAGC
	R: TGGCAAGACCTTACG-GACATCT
GAPDH	F: AGATCATCAGCAATGCCTCCT
	R: TGAGTCCTTCCACGATACCAA

### Co-culture and immune cell assays

A Transwell co-culture system was used to study the interactions between prostate cancer cells and macrophages. LDH activity and ELISA assessed immune cell-mediated cytotoxicity and cytokine secretion. Flow cytometry was used for immune cell sorting and analysis. The detailed methods are included in the supplementary materials.

### Subcutaneous tumor xenograft model

For the *in vivo* study, 12 female C57BL/6 mice (4–5 weeks old, 18–20 g) were obtained from the Shanghai Experimental Animal Center and maintained under specific pathogen-free (SPF) conditions. Mice were housed at 22 ± 2 ℃, 50 ± 10% relative humidity, with a 12-hour light/dark cycle and free access to food and water. After a one-week acclimation period, the experiment was initiated. A total of 1 × 10^6^ TRAMP-C1 cells (stably transfected with sh-NC or sh-TMSB10) were suspended in 200 μL HEPES-buffered saline (HBS, Gibco, USA) and subcutaneously injected into the right axilla using a 1 mL syringe (BD Biosciences, USA). The injection was performed slowly to ensure uniform distribution, followed by a gentle massage of the injection site. Tumor growth was monitored daily, recording size, appearance, and overall health status. Every two days, the longest diameter (D) and shortest diameter (d) were measured using a vernier caliper, and tumor volume was calculated as D × d^2^/2. After 25 days, mice were euthanized via cervical dislocation, and tumors were immediately excised and weighed using an electronic balance (Mettler Toledo, Switzerland). Tumor tissues were then fixed in 10% neutral-buffered formalin (Sigma-Aldrich, USA) for histological analysis. All procedures were conducted in strict accordance with institutional animal ethics guidelines.

### Statistical analysis

In this study, we employed various precise statistical methods to ensure scientific rigor. For bioinformatics analyses, R software (version 4.0.2) was primarily used for data processing and analysis. The relationship between TMSB10 and clinical pathological features was assessed using Kruskal-Wallis and Wilcoxon signed-rank tests. Survival analyses included Cox proportional hazards regression models to estimate the effects of various factors on OS, with survival curves drawn using the Kaplan-Meier method.

In the statistical analysis of cell experiments, we analyzed differences and relationships between experimental groups. For cell migration and invasion assays, t-tests or one-way ANOVA were used to compare the number of migrating and invading cells between different groups. In cell cycle and apoptosis analyses, Chi-square tests or Fisher’s exact tests were used to analyze differences in cell distribution at various stages. For experiments involving multiple time points or doses, repeated measures ANOVA or mixed-effects models were used to analyze the effects of time or dose and their interaction with treatment groups. All cell experiments were repeated at least three times to ensure the reliability of the results. Throughout the analysis, P < 0.05 was used as the criterion for statistical significance, while P < 0.01 was considered to indicate statistically solid significance.

## Results

### Expression of TMSB10 in various cancer types and its association with TMB and MSI

**Fig. 1:**
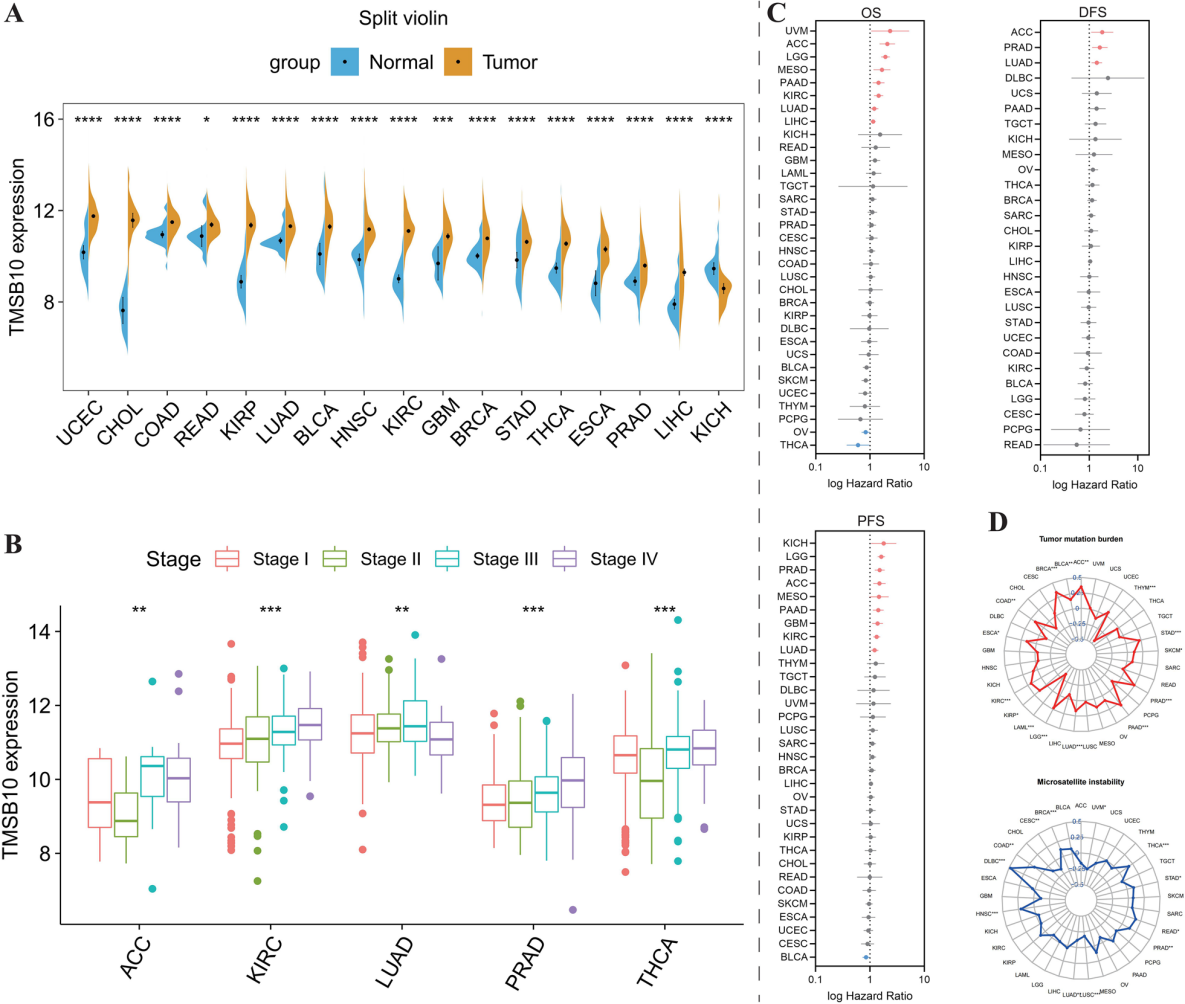
Distribution of TMSB10 expression in various cancers and its correlation with tumor grading and prognosis. **A** Depicts TMSB10 expression levels in various cancer types and their corresponding normal tissues. **B** Correlation of TMSB10 expression with tumor grading. **C** Univariate Cox regression analysis of TMSB10 as a prognostic factor. **D** Correlation between TMSB10 expression and TMB & MSI in different cencers. Asterisks indicate levels of statistical significance (*P < 0.05, **P < 0.01, ***P < 0.001, ****P < 0.0001)

This study initially analyzed the differential expression of TMSB10 in TCGA pan-cancer samples compared to corresponding normal tissues. As shown in Figure 1A, TMSB10 was upregulated in most cancer types compared to normal tissues, including bladder cancer, breast cancer (BRCA), colon adenocarcinoma (COAD), glioblastoma multiforme, head and neck squamous cell carcinoma, kidney renal clear cell carcinoma (KIRC), lung adenocarcinoma (LUAD), lung squamous cell carcinoma, prostate adenocarcinoma (PRAD), rectal adenocarcinoma, stomach adenocarcinoma, thyroid carcinoma (THCA), and uterine corpus endometrial carcinoma. However, in kidney chromophobe (KICH), TMSB10 expression was significantly higher in normal tissues than in other cancer types (P < 0.0001). Additionally, in KIRC, PRAD, and THCA patients, a positive correlation was observed between TMSB10 expression levels and tumor grading improvement (P < 0.001, Figure 1B). Univariate Cox regression analysis further confirmed the value of TMSB10 as a prognostic factor (Figure 1C). High TMSB10 expression indicated poor prognosis in adrenal cortical carcinoma and LUAD patients and a significant association with worse DFS and PFS in PRAD patients. In cancers like BRCA, COAD, LUAD, and PRAD, TMSB10 expression positively correlated with TMB and MSI (Figure 1D).

### High TMSB10 expression correlates with poor prognosis in prostate cancer

**Fig. 2:**
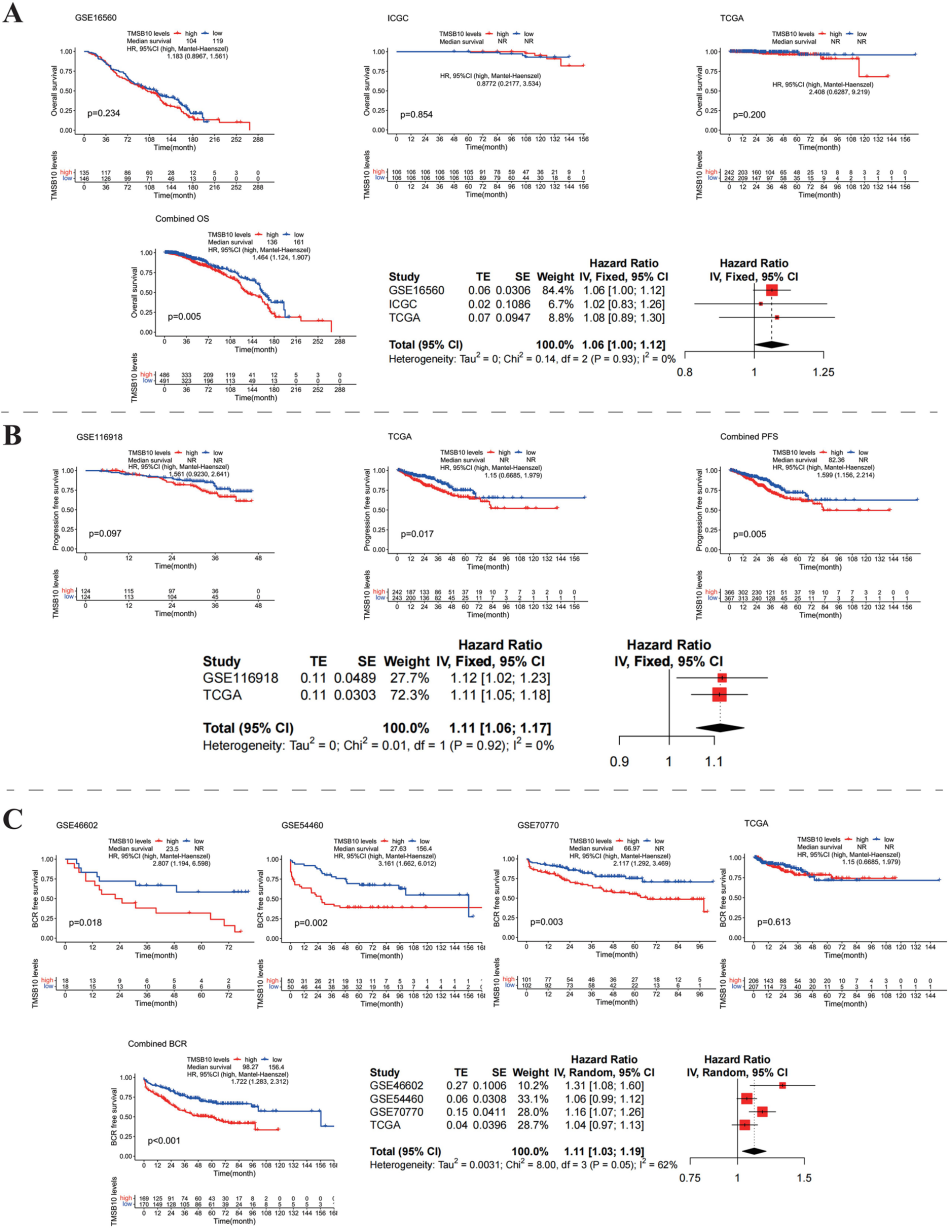
Multi-cohort analysis of the relationship between TMSB10 expression and prognosis in prostate cancer. **A** Survival curves correlating high TMSB10 expression with poor OS prognosis in different independent cohorts (GSE16560, ICGC, and TCGA). **B** Impact of high TMSB10 expression on PFS in prostate cancer patients. **C** Relationship between TMSB10 expression and biochemical recurrence (BCR) free survival in GSE46602, GSE5440, GSE70770, and TCGA cohorts. HRs and 95% CIs below each section reveal the magnitude of the combined effect obtained through meta-analysis, indicating high TMSB10 expression as a significant indicator of poor prognosis. Asterisks denote levels of statistical significance (*P < 0.05, **P < 0.01, ***P < 0.001)

PCA analysis confirmed the normalization of gene expression across multiple cohorts (Figure S1A, B). In prostate cancer cohorts using overall survival (OS) as an endpoint, high TMSB10 expression was associated with a trend toward poorer prognosis in GSE16560, ICGC, and TCGA cohorts (Figure 2A), though limited by small sample sizes and the slow progression of prostate cancer. However, pooled data revealed a significant association between high TMSB10 expression and shorter OS (P = 0.005, HR = 1.464, 95% CI [1.12, 1.9]), with meta-analysis confirming its role as an adverse risk factor (fixed-effect model, HR = 1.06, 95% CI [1.00, 1.12]).

Using PFS as the endpoint, high TMSB10 expression was associated with poorer prognosis in GSE116918 and TCGA cohorts (P = 0.097, 0.017, respectively, Figure 2B). Combined cohort analysis further confirmed the association (P = 0.005, HR = 1.599, 95% CI [1.156, 2.214]), with meta-analysis supporting increased risk (fixed-effect model, HR = 1.11, 95% CI [1.06, 1.17]). Analysis using biochemical recurrence-free survival (BCR-FS) as an endpoint yielded similar findings. High TMSB10 expression predicted poor prognosis across most cohorts (P = 0.018, 0.002, 0.003, 0.613, Figure 2C), with integrated analysis (P < 0.001, HR = 1.722, 95% CI [1.283, 2.312]) and meta-analysis (random-effects model, HR = 1.11, 95% CI [1.03, 1.19]) further supporting its prognostic significance.

### Association of TMSB10 expression with prostate cancer grading

**Fig. 3:**
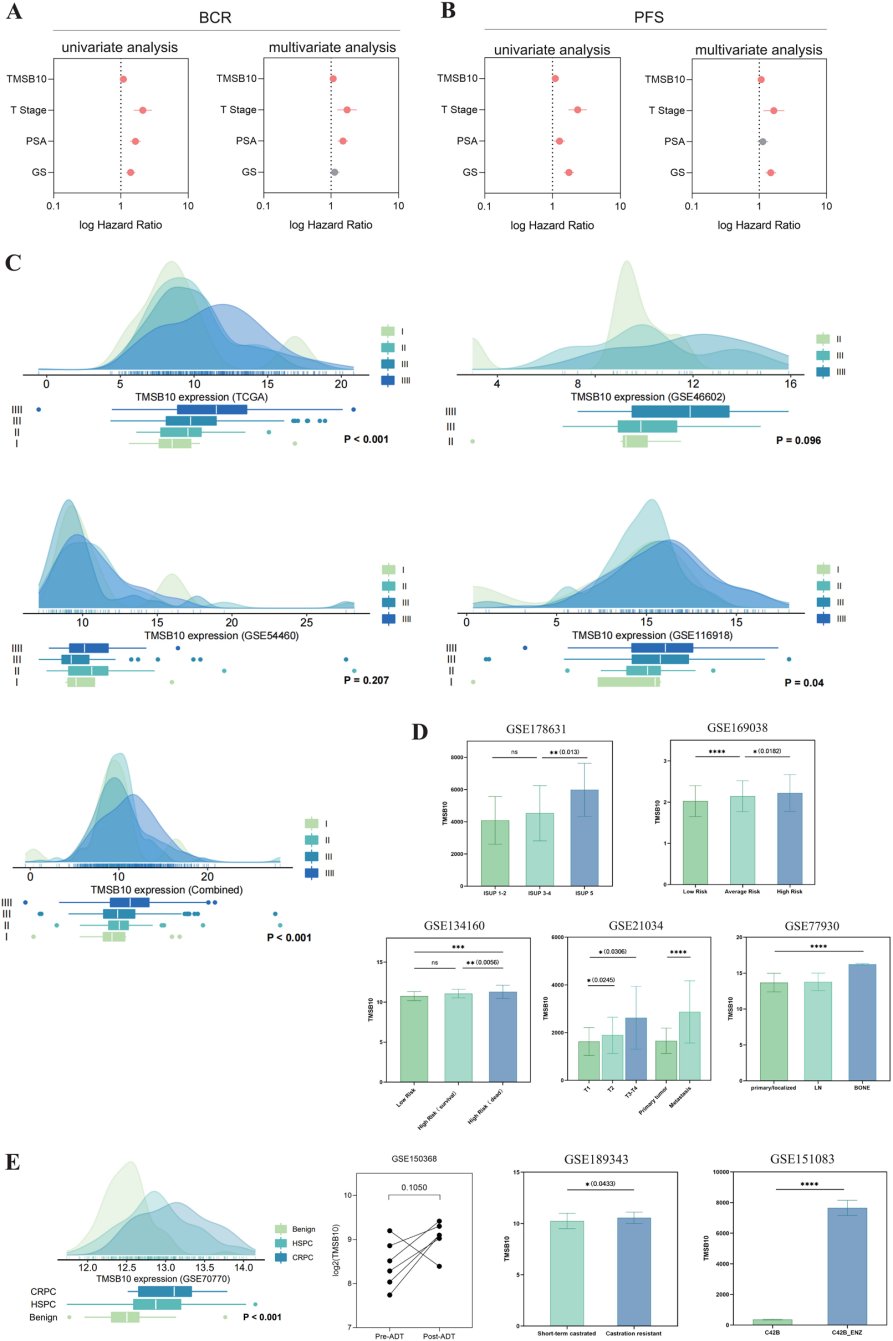
Analysis of the correlation between TMSB10 expression and prostate cancer prognostic indicators. **A** Univariate and multivariate Cox regression analysis results showing the correlation of TMSB10, tumor stage, prostate-specific antigen (PSA) levels, and Gleason score (GS) with biochemical recurrence (BCR) risk. **B** Univariate and multivariate analysis results for progression-free survival (PFS). **C** Density distribution plots of TMSB10 expression across different datasets (TCGA and GEO studies) illustrate its association with prostate cancer staging. **D** Bar graphs depicting differences in TMSB10 expression across prostate cancer patient groups in different risk categories. **E** TMSB10 expression about pre-and post-treatment states of prostate cancer (ADT treatment). Asterisks denote statistical significance (*P < 0.05, **P < 0.01, ***P < 0.001)

Table 4 summarizes the clinical differences between high and low TMSB10 expression groups. Patients in the high-expression group had significantly higher tumor age, PSA, GS, and T-stage (P < 0.001, 0.013, < 0.001, < 0.001, respectively), along with increased tumor metastasis and positive surgical margins (P = 0.04, 0.031). Univariate and multivariate analyses confirmed TMSB10 as an independent prognostic factor for BCR and PFS (Fig. 3A, B).

**Table 4:** Association between TMSB10 expression and clinicopathological parameters (pooled from different cohort) of patients with prostate cancer

	High expression	Low expression	P-value
Age, median (IQR) (H: L = 736: 736)	65 (59, 72)	64 (58, 69)	<0.001
PSA, median (IQR) (H: L = 531: 531)	9.4 (6.3, 17.5)	8.5 (5.7, 14.5)	0.013
GS (H: L = 675: 675)			<0.001
<7	95 (14.1%)	144 (21.3%)	
7	324 (48.0%)	365 (54.1%)	
8	95 (14.1%)	81 (12.0%)	
>8	161 (23.9%)	85 (12.6%)	
T (H: L = 623: 624)			<0.001
≤T2	306 (49.1%)	399 (63.9%)	
T3	308 (49.4%)	218 (34.9%)	
T4	9 (1.4%)	7 (1.1%)	
N (H: L = 260: 260)			0.096
N1	50 (19.2%)	35 (13.5%)	
N0	210 (80.8%)	225 (86.5%)	
M (H: L = 241: 242)			0.004
M1	8 (3.3%)	0 (0.0%)	
M0	233 (96.7%)	242 (100.0%)	
PSM (H: L = 119: 120)			0.031
Y	50 (42.0%)	34 (28.3%)	
N	69 (58.0%)	86 (71.7%)	
Fusion (H: L = 135: 137)			1
Y	23 (17.0%)	23 (16.8%)	
N	112 (83.0%)	114 (83.2%)	

The Fisher’s exact test was performed to analyze the differences in different clinical characteristics in high- and low- TMSB10 expression groups

*PSA* prostate specific antigen, *GS* Gleason score, *PSM* positive surgical margins

**Fig. 4:**
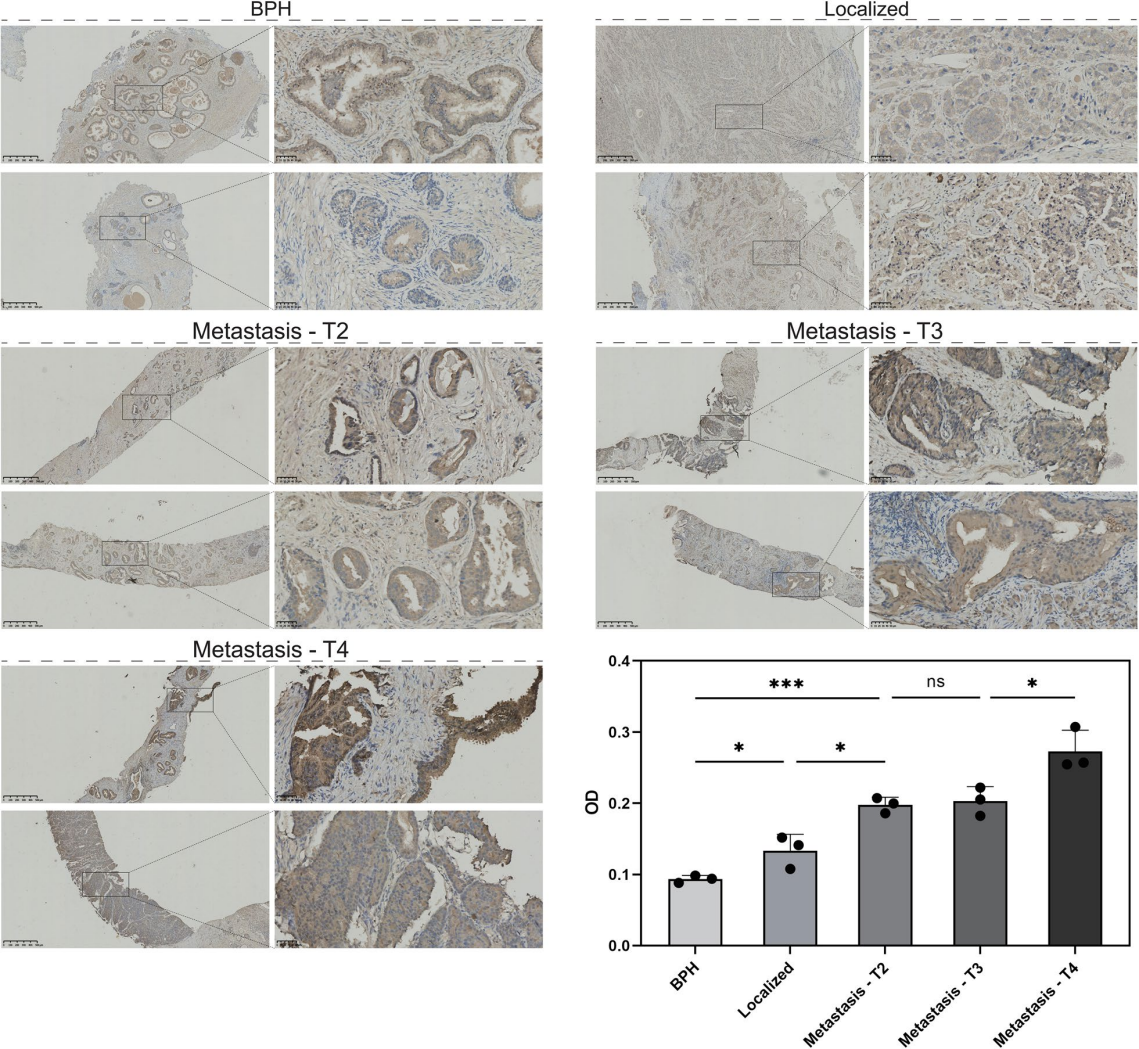
Immunohistochemical expression patterns of TMSB10 in prostate cancer tissue grading and metastatic states. A progressive increase in TMSB10 staining intensity is observed from Benign Prostatic Hyperplasia (BPH) to Localized prostate cancer and through various stages of metastatic prostate cancer (T2 to T4). The histogram in the lower right quantitatively displays the comparison of OD values of TMSB10 expression in different tissue types, with asterisks indicating statistical significance (*P < 0.05, **P < 0.01, ***P < 0.001, ns represents non-significance)

Higher TMSB10 expression correlated with increased EAU risk groups (Fig. 3C), showing significant differences in TCGA and multiple datasets (P < 0.001, 0.096, 0.207, 0.004), with validation in combined cohorts (P < 0.001) and other public datasets (Fig. 3D). Further analysis across GSE178631, GSE169038, GSE134160, GSE21034, and GSE77930 revealed significant upregulation of TMSB10 in high ISUP grade and advanced T-stage prostate cancers, as well as metastatic cases. As Fig. 3E showed, progressive TMSB10 upregulation was observed in benign, HSPC, and CRPC groups (GSE70770, P < 0.001) and showed an increasing trend post-ADT treatment (GSE150368, P = 0.105). Long-term castrated patients (GSE189343) exhibited significantly higher TMSB10 levels compared to short-term (P = 0.0433), and enzalutamide-resistant C42B cells (GSE151083) also displayed upregulation. Immunohistochemical analysis confirmed these findings (Fig. 4), showing a progressive increase in TMSB10 expression from BPH to localized and metastatic high-grade prostate cancer, with significantly higher levels in metastatic cases (P < 0.0001).

### High TMSB10 expression associated with immune suppression and immune evasion pathways

**Fig. 5:**
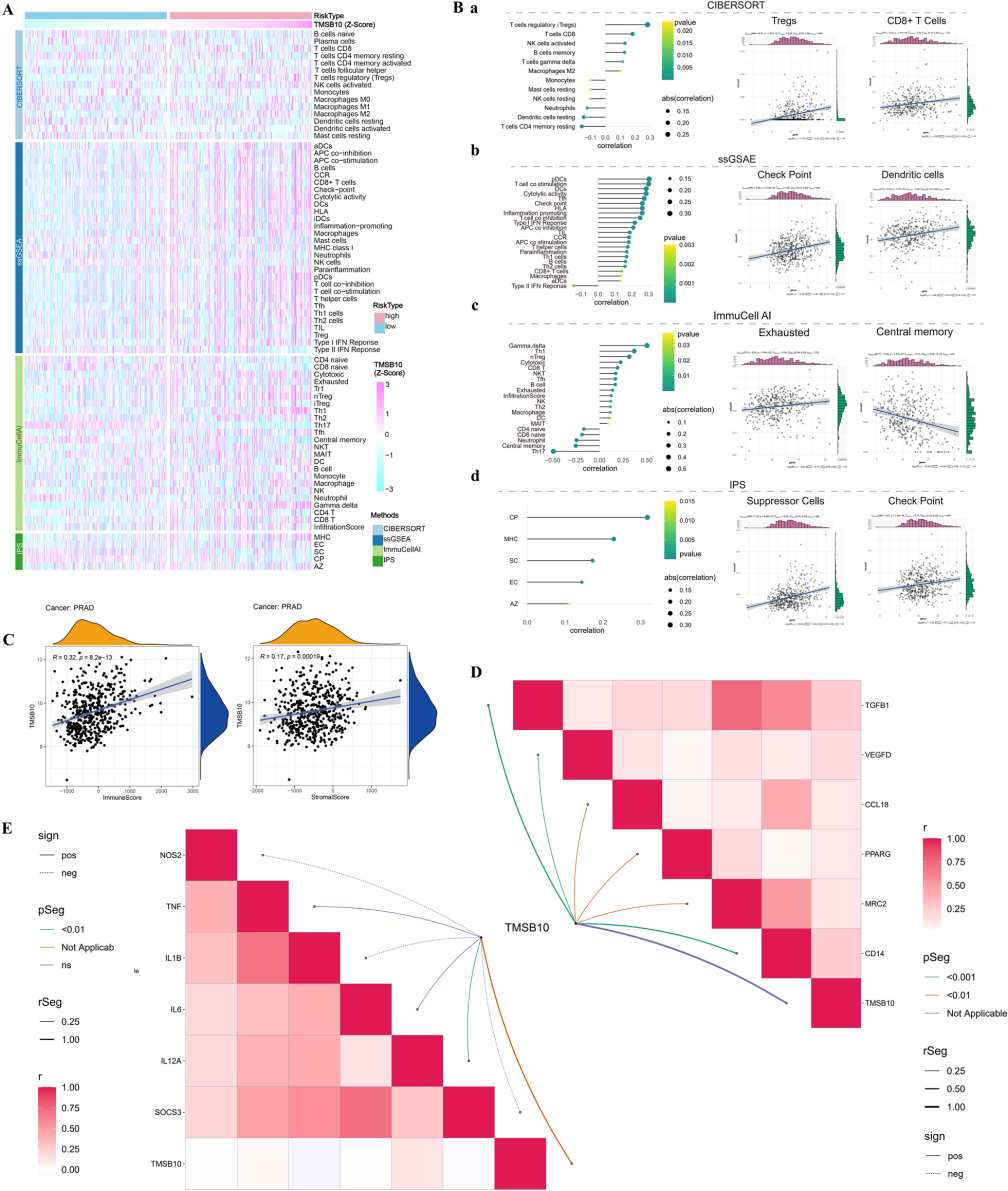
Analysis of the association between high TMSB10 expression and immune suppression. **A** Displays the immunological landscape calculated for different TMSB10 expression groups in the TCGA cohort, analyzed using four algorithms. **B** A lollipop plot shows each algorithm’s assessment of immune infiltration, indicating an association between high TMSB10 expression and immune suppression. **C** Correlation between TMSB10 expression and immune and stromal scores. **D**, **E** Show a robust positive correlation between TMSB10 expression and gene expression related to M2 macrophages and a negative correlation with M1 macrophages, suggesting a shift from M1 to M2 macrophages in cancers with high TMSB10 expression

The heatmap in Fig.5A illustrates the immune landscape of different TMSB10 expression groups in the TCGA cohort, calculated using four algorithms. Higher TMSB10 Z-Scores correlated with increased immune-related gene expression. The lollipop plot further confirmed that high TMSB10 expression is associated with immune suppression, characterized by CD8+ T cell infiltration, enhanced antigen-presenting activity, and increased Treg enrichment and immune checkpoint expression (Figure 5B). Despite immune activation, immunosuppressive mechanisms were predominant in the high TMSB10 group, suggesting a complex immune regulatory environment.

Immune and stromal scores in Figure 5C showed a positive correlation with TMSB10 expression, with a stronger association with immune scores, supporting the accuracy of the immune microenvironment analysis. In macrophage polarization analysis (Figure 5D, E), TMSB10 was positively correlated with M2-related gene expression and negatively correlated with M1-related genes, indicating a potential shift from M1 to M2 macrophages in tumors with high TMSB10 expression.

TMSB10 overexpression was linked to immune suppression, including increased immune checkpoint expression (Figure S2A). In vitro experiments demonstrated that TMSB10 knockdown reduced tumor cell surface immune checkpoint levels (Figure S2B), further confirmed by immunofluorescence analysis (Figure S2C). IMvigor210 analysis (Figure S2D) suggested that higher TMSB10 levels were associated with a stronger immunotherapy response. Stratification by CTLA4/PD1 expression (Figure S2E) showed significantly higher immune scores in the high TMSB10 group, except in CTLA4-/PD1- cases, where no significant difference was observed.

### TMSB10 silencing inhibits proliferation, migration, and invasion of LNCaP and DU145 Cells

**Fig. 6:**
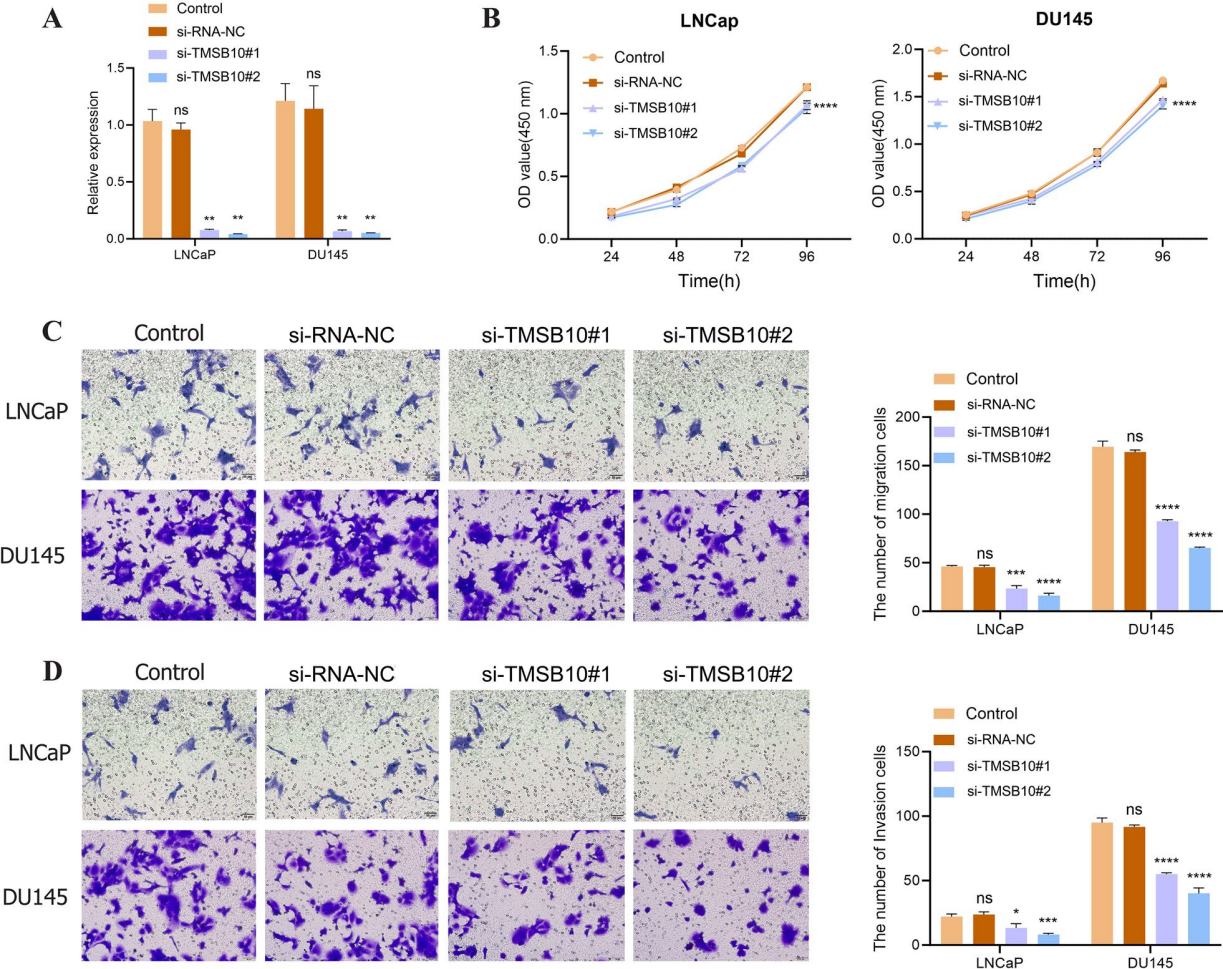
TMSB10 silencing inhibits prostate cancer cell proliferation, migration, and invasion. **A** TMSB10 mRNA expression levels in different cell lines after transfection with si-TMSB10#1 and si-TMSB10#2 (vs. Control group, P < 0.01). **B** CCK-8 assay results showing cell proliferation after transfection with si-TMSB10#1 and si-TMSB10#2 (vs. Control group, ****P < 0.0001,*P < 0.001). **C**, **D** Transwell cell migration assay results (compared to the control group, ****P < 0.0001; ***P < 0.001; *P < 0.05). All experiments were repeated three times

QRT-PCR results confirmed that si-TMSB10#1 and si-TMSB10#2 significantly reduced TMSB10 expression compared to the control and NC groups (Figure 6A). Both siRNAs effectively silenced TMSB10, eliminating potential off-target effects, with si-TMSB10#2 showing higher silencing efficiency.

Consistent with expectations, CCK-8 assays demonstrated that TMSB10 knockdown significantly suppressed proliferation in both cell lines (Figure 6B). Further analysis of cell migration and invasion revealed that TMSB10 knockdown significantly reduced the number of migrating LNCaP and DU145 cells, as shown in Figure 6C. Similarly, invasion assays showed fewer siRNA-treated cells penetrating the matrix compared to controls (Figure 6D), with statistically significant differences.

### Overexpression of TMSB10 promotes proliferation, migration, and invasion in LNCaP and DU145 cells

**Fig. 7:**
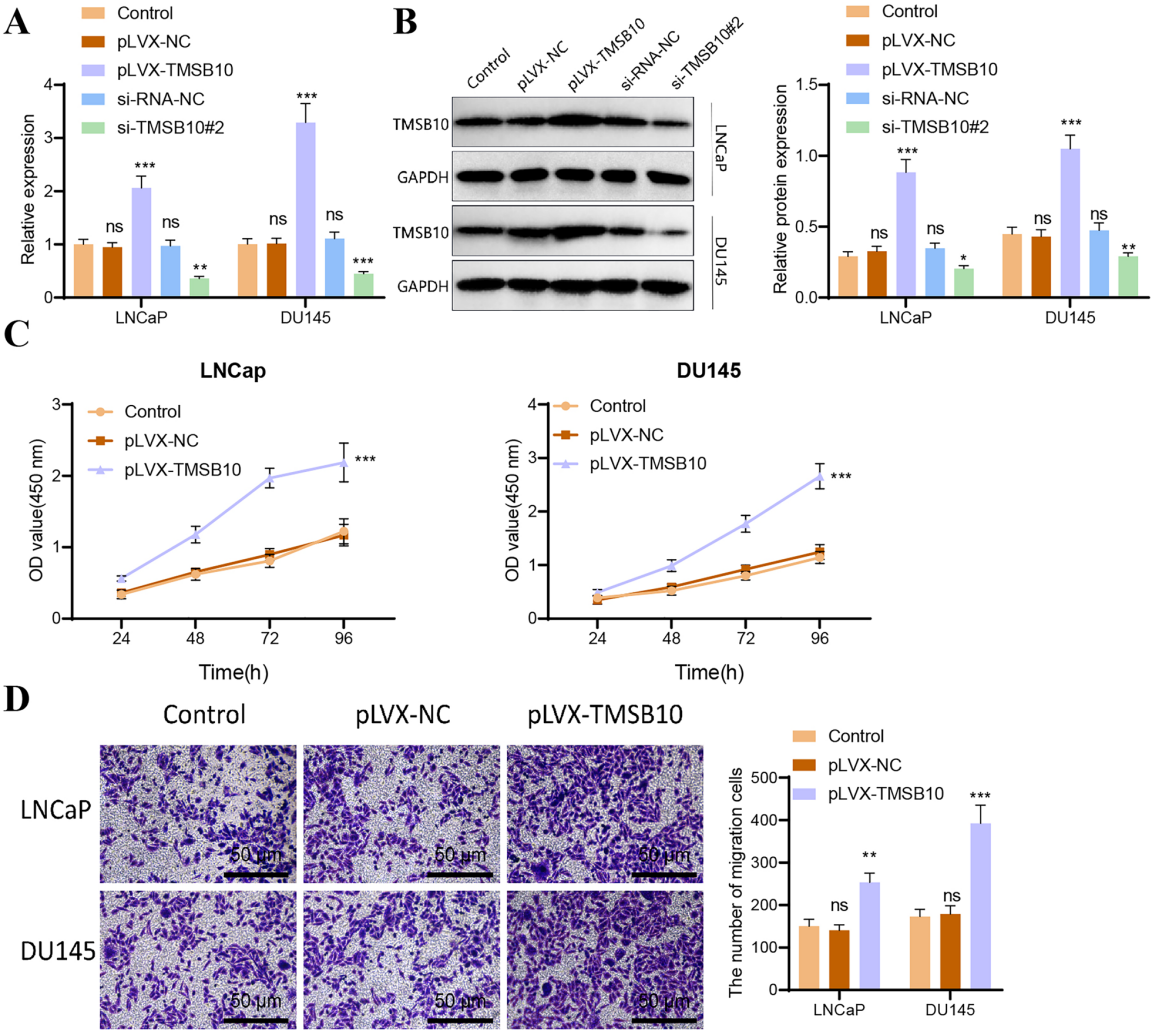
TMSB10 overexpression promotes proliferation, migration, and invasion in prostate cancer cells. **A** Expression levels of TMSB10 mRNA in different cell lines following overexpression and silencing (compared to the control group, ***P < 0.001; **P < 0.01; ns: non-significant). **B** Protein expression levels in different cell lines post-TMSB10 overexpression and silencing (compared to the control group, ***P < 0.001; **P < 0.01; *P < 0.05; ns: non-significant). **C** CCK-8 cell proliferation assay results in post-TMSB10 overexpression (compared to the control group, ***P < 0.001). **D** Transwell cell migration assay results (compared to the control group, ***P < 0.001; **P < 0.01). All experiments were repeated three times

QRT-PCR results showed that TMSB10 expression was significantly elevated in the overexpression group compared to the control and NC groups (Figure 7A). Western blot results (Figure 7B) also indicated a significant increase in TMSB10 protein expression in the overexpression group. Consistent with expectations, CCK-8 cell proliferation assays (Figure 7C) demonstrated that overexpression of TMSB10 significantly enhanced the proliferation capacity of both cell types compared to the control group. We further explored the effects of TMSB10 expression on cell migration and invasion. As shown in Figure 7D, TMSB10 overexpression facilitated cell migration, significantly increasing the number of LNCaP and DU145 cells migrating from the upper to the lower chamber.

### Silencing of TMSB10 inhibits cell cycle progression and promotes cell apoptosis

**Fig. 8:**
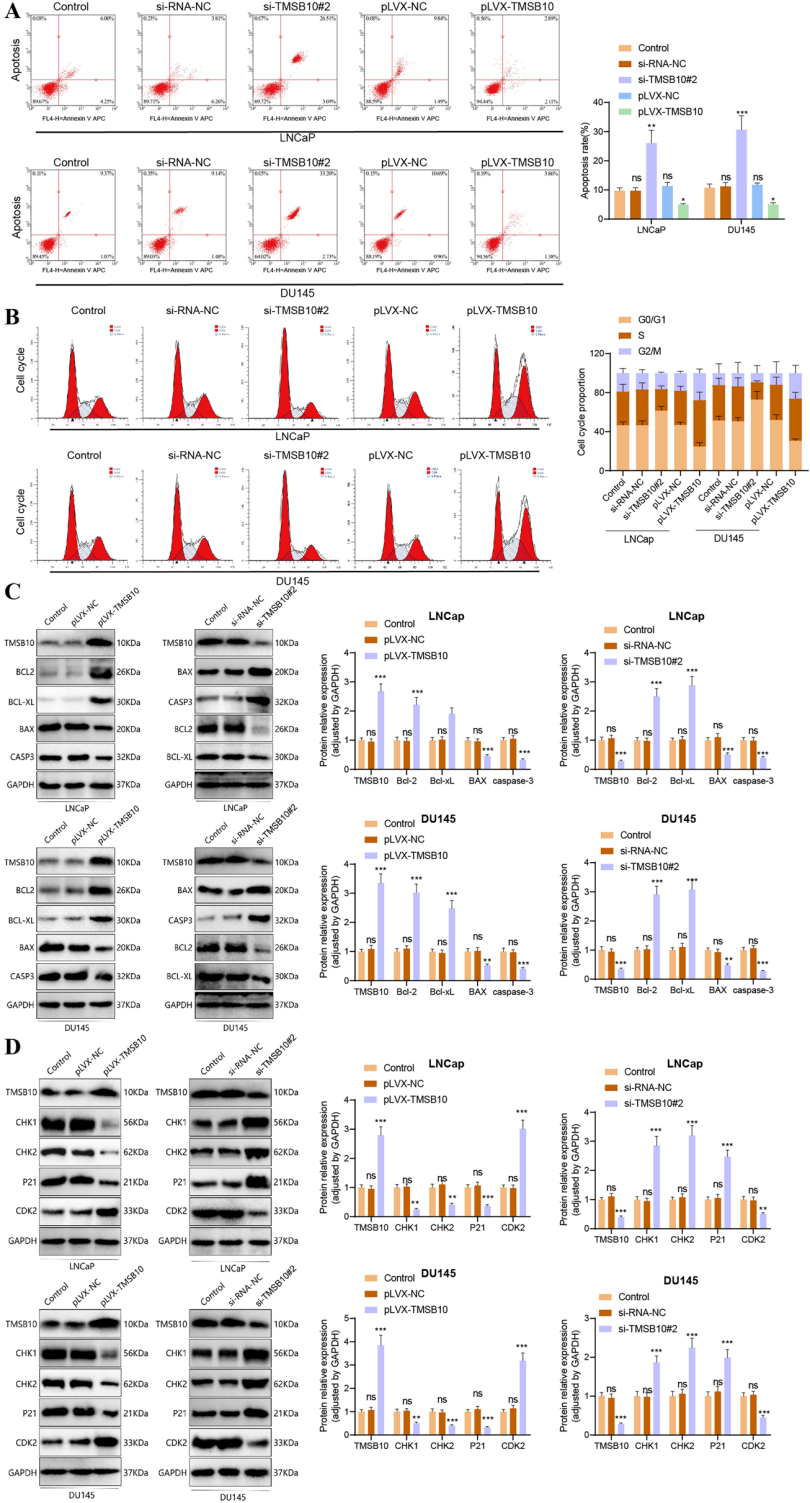
Regulatory effects of TMSB10 expression on the cell cycle progression and apoptosis in prostate cancer cells. **A** Flow cytometry analysis showing apoptosis rates in LNCaP and DU145 cells (compared to the control group, ***P < 0.001; **P < 0.01; *P < 0.05; ns: non-significant). **B** Analysis of cell cycle progression. **C** Western blot analysis further explores the molecular mechanisms of apoptosis. **D** Western blot analysis investigating the specific molecular mechanisms of the cell cycle (compared to the control group, ****P < 0.0001; ***P < 0.001; **P < 0.01; ns: non-significant). All cell experiments were repeated three times

Flow cytometry analysis demonstrated that TMSB10 knockdown (si-TMSB10#2) increased apoptosis in LNCaP and DU145 cells, while TMSB10 overexpression reduced apoptosis (Figure 8A). Cell cycle analysis revealed a significant increase in S-phase cells following TMSB10 overexpression, suggesting enhanced proliferation, whereas TMSB10 knockdown reversed this effect (Figure 8B).

Western blot analysis further confirmed these findings (Figure 8C, D). TMSB10 overexpression reduced pro-apoptotic proteins (BAX, caspase-3) while increasing anti-apoptotic proteins (Bcl-2, Bcl-xL). Additionally, cell cycle inhibitors (CHK1, CHK2, p21) were downregulated, whereas CDK2 was upregulated, supporting the role of TMSB10 in promoting cell cycle progression and inhibiting apoptosis.

### Overexpression of TMSB10 promotes tumor cell immune infiltration and immune evasion

**Fig. 9:**
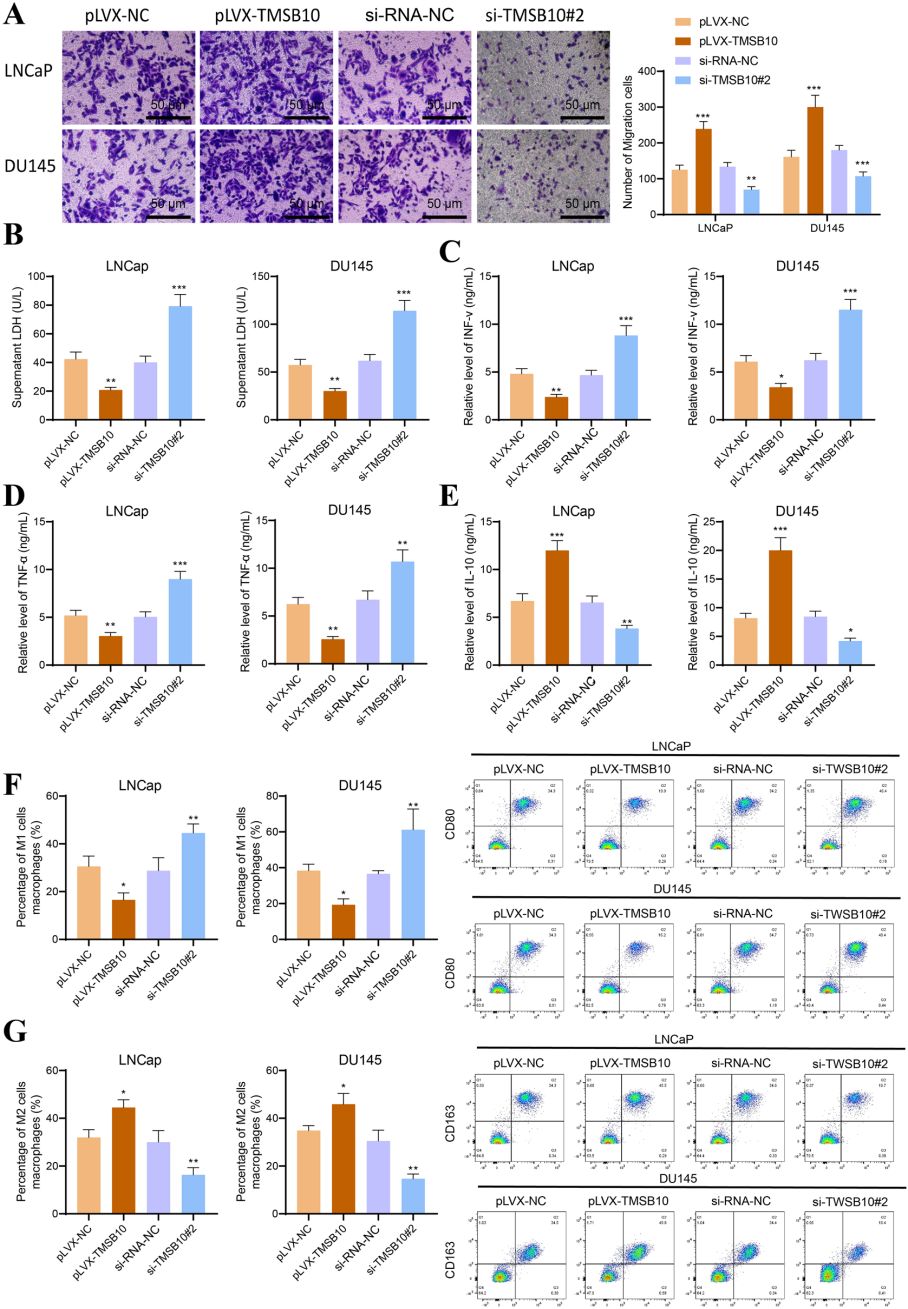
Effects of TMSB10 regulation on immune cell function and migration ability on tumor cells. **A** Transwell assay assessing immune cell migration capability in different co-culture systems. **B** LDH release assay detects immune cells’ cytotoxic effect on LNCaP or DU145 cells in various co-culture systems. **C–E** ELISA tests the expression levels of INF-γ, TNF-α, and IL-10 secreted by immune cells in different co-culture systems. **F** Flow cytometry analysis identifying M1-type macrophages using CD80/CD86 markers. **G** Flow cytometry analysis identifying M2-type macrophages using CD163/CD206 markers (compared to the NC group, ***P < 0.001, **P < 0.01, *P < 0.05). All cell experiments were repeated three times

Transwell assays demonstrated that macrophage migration increased after co-culture with si-TMSB10#2-treated LNCaP or DU145 cells, whereas pLVX-TMSB10-treated cells significantly reduced macrophage migration (Figure 9A).

LDH release assays assessed macrophage cytotoxicity against tumor cells. Macrophages co-cultured with pLVX-TMSB10-treated cells showed reduced cytotoxicity, while those co-cultured with si-TMSB10#2-treated cells exhibited enhanced tumor-killing activity. Notably, in the absence of macrophage co-culture, neither pLVX-TMSB10 nor si-TMSB10#2 directly affected LNCaP or DU145 cell viability (Figure 9B).

ELISA results revealed changes in cytokine secretion. Macrophages co-cultured with pLVX-TMSB10-treated cells secreted low rIFN-γ and TNF-α but higher IL-10, while those co-cultured with si-TMSB10#2-treated cells showed increased IFN-γ and TNF-α with reduced IL-10 (Figure 9C–E). Flow cytometry analysis indicated macrophage subtype shifts. Co-culture with pLVX-TMSB10-treated cells decreased M1 macrophages and increased M2 macrophages, whereas si-TMSB10#2-treated cells induced the opposite effect (Figure 9F, G).

### TMSB10 knockdown inhibits prostate cancer growth in vivo

**Fig. 10:**
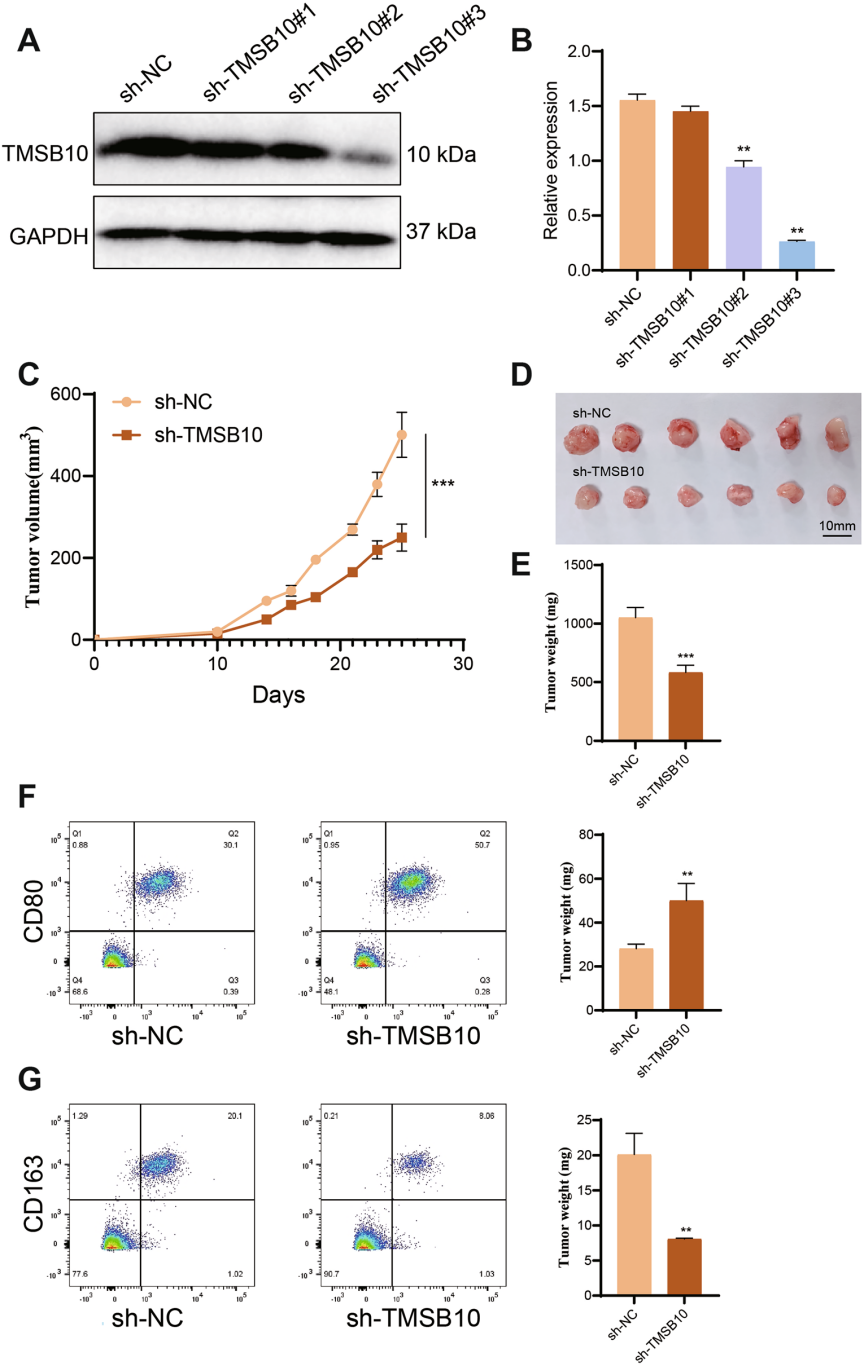
Effect of TMSB10 on prostate cancer xenograft growth in mice. **A** Western blot analysis of TMSB10 protein expression in DU145 cell samples. **B** Quantification of Western blot results. **C** Tumor growth curve of xenografts in sh-NC and sh-TMSB10 groups. **D** Representative images of subcutaneous prostate cancer xenografts in mice. **E** Tumor weight analysis in different groups. **F** Flow cytometry analysis of CD80/CD86 markers for identifying M1 macrophages. **G** Flow cytometry analysis of CD163/CD206 markers for identifying M2 macrophages. Data are presented as mean ± SD. Statistical significance compared to the sh-NC group: ***P < 0.001, **P < 0.01, *P < 0.05

A subcutaneous xenograft model was established using DU145 cells stably transfected with sh-TMSB10 or sh-NC. Among the three constructs, sh-TMSB10#3 exhibited the most efficient silencing and was selected for further experiments (Figure 10A, B). TMSB10 knockdown significantly reduced tumor volume and weight compared to the sh-NC group, indicating its role in promoting tumor growth (Figure 10C–E). Flow cytometry analysis further showed an increase in M1 macrophages and a decrease in M2 macrophages in the sh-TMSB10 group, suggesting a shift towards an anti-tumor immune response (Figure 10F, G).

## Discussion

The thymosin β family (including TMSB10, TMSB4, and TMSB15) plays a significant role in cellular biological processes such as proliferation, differentiation, repair, and apoptosis (Pan et al. 2020). Previous studies have shown that TMSB15 and TMSB4 are closely associated with the migration of prostate cancer cells, but research on the expression and mechanism of TMSB10 in prostate cancer has been relatively limited (Choi et al. 2023; Faa et al. 2021). Building on this, our study delves into the role of TMSB10 in prostate cancer through bioinformatics, meta-analysis, and immunohistochemistry experiments.

Our research confirms the strong correlation of high TMSB10 expression with clinical tumor grading in prostate cancer and its role as an independent predictor of poor prognosis. TMSB10 plays a pivotal role in modulating the immune microenvironment, a function that aligns with and extends beyond previous research findings (Fang et al. 2022; Li et al. 2023). Compared to earlier studies, which predominantly focused on TMSB10’s expression patterns across various cancers and their correlation with tumor progression (Pan et al. 2020), our study provides a more in-depth mechanistic explanation and novel insights. Previous reports on how TMSB10 influences the immune microenvironment, particularly its effect on macrophage polarization, have been limited. M2-type macrophages, known as immunosuppressive macrophages, are generally associated with tumor promotion, immune escape, and maintaining an inflammatory environment (Shu et al. 2022). Prior research primarily regarded TMSB10’s function as directly facilitating tumor cell proliferation, invasion, and metastasis (Zeng et al. 2020). However, understanding of TMSB10’s impact on the tumor microenvironment, especially on the functionality of immune cells, remains relatively limited. Through a comprehensive approach, including immunohistochemistry, flow cytometry, and gene expression analysis, our study delves into how TMSB10 promotes prostate cancer development by affecting macrophage polarization. We observed a significant increase in the proportion of M2-type macrophages in prostate cancer tissues with high TMSB10 expression, suggesting TMSB10’s direct or indirect involvement in regulating macrophage polarization. This finding offers a new perspective on TMSB10’s complex role in tumor progression.

Furthermore, our experimental data indicate that TMSB10 can enhance the proliferation and survival of M2-type macrophages. This effect is likely mediated by influencing the expression of cell cycle-related genes, such as cyclin D1 and cyclin E (Lin et al. 2006), and by inhibiting the expression of apoptosis-related genes. These findings provide a fresh viewpoint on how TMSB10 may influence tumor growth and progression by modulating the functions of immune cells.

This study identifies TMSB10 overexpression as an adverse prognostic factor in prostate cancer, suggesting its potential as a biomarker for clinical stratification, prognosis assessment, and personalized therapy. High TMSB10 expression correlates with increased tumor aggressiveness, immune evasion, and treatment resistance, underscoring its role in disease progression. TMSB10 plays a crucial role in immune regulation and is strongly associated with PD-1/PD-L1 and CTLA-4 checkpoint expression (Li et al. 2022a, b). It may facilitate immune evasion by altering the tumor microenvironment, notably through M2 macrophage polarization and secretion of immunosuppressive factors such as IL-10 and TGF-β (Han et al. 2022). Additionally, its involvement in cytoskeletal remodeling (Zhang et al. 2018) may influence tumor-immune cell interactions, further modulating immune checkpoint expression (Zeng et al. 2020).

Given its role in tumor progression and immune resistance, TMSB10 represents a potential therapeutic target. Targeting TMSB10 could inhibit tumor cell migration and invasion while enhancing the immune response. Although no specific small-molecule inhibitors or monoclonal antibodies targeting TMSB10 are currently available, their development could improve immune checkpoint inhibitor efficacy (e.g., PD-1/PD-L1 inhibitors) and lead to combination therapy strategies. This study provides a theoretical basis for targeting TMSB10 and highlights its potential in immunotherapy-based treatment approaches. Future research should elucidate TMSB10’s molecular mechanisms and assess its clinical viability in prostate cancer immunotherapy.

## Conclusion

This study identifies a correlation between TMSB10 and immune evasion, yet its precise molecular mechanisms require further experimental validation. While TMSB10’s role in immune microenvironment regulation has been observed, its direct impact on immune checkpoint expression and evasion remains unclear. Additionally, TMSB10 functions may vary across different cancer types, and its broader oncogenic role warrants further investigation. A key limitation is the heterogeneity of public datasets, where factors such as treatment history, ethnicity, and age may introduce bias in TMSB10 expression patterns and tumor progression correlations (Vadaq et al. 2022). Despite applying multilayer statistical corrections to minimize confounding variables, residual bias may still affect the generalizability of findings.

Future studies will employ gene knockout and overexpression models alongside immunohistochemistry, flow cytometry, and co-immunoprecipitation to elucidate TMSB10’s role in tumor immune microenvironment regulation. The focus will be on TMSB10-immune checkpoint interactions to uncover its regulatory network. Future research will expand to other cancer types to account for inter-tumor heterogeneity to assess TMSB10 as a pan-cancer therapeutic target. Additionally, future studies will incorporate rigorous clinical and experimental design, cross-platform validation, and stratified analyses to address dataset variability to improve reliability and reproducibility. Ultimately, in vitro, *in vivo*, and clinical sample validation will be essential to confirming TMSB10’s functional role in prostate cancer and beyond.

## Supplementary Information

Additional file 1: Figure S1. PCA analysis of gene expression normalization across multiple cohorts. Note:Raw PCA analysis of the combined expression profile across multiple cohorts, showing the initial variance before normalization.PCA analysis after applying the Combat algorithm to normalize gene expression across cohorts, demonstrating that gene expression levels have been successfully corrected to a similar level

Additional file 2: Figure S2. Analysis of the association between TMSB10 expression, immune checkpoints, immunofluorescence, and response to immunotherapy. Note:Demonstrates the expression of immune checkpointsin the high TMSB10 group in the TCGA cohort.Interfering with TMSB10 expression significantly reduces the expression levels of tumor cell surface immune checkpoints.Immunofluorescence testing results show enhanced fluorescence signals of immune checkpoints in TMSB10 high-expression sections.Based on the IMvigor210 database, increased TMSB10 expression levels are associated with a more pronounced response to immunotherapy.Comparison of immune-related scores among CTLA4 and PD1 groups. Asterisks denote statistical significance

Additional file 3.

Additional file 4.

Additional file 5.

Additional file 6.

Additional file 7.

Additional file 8.

Additional file 9.

## Data Availability

All data can be provided as needed.
