# Supplementary figures and images for: TMSB10 drives prostate cancer aggressiveness via immune microenvironment regulation
Source: Mol Med. 2025 Apr 30;31:160. doi: 10.1186/s10020-025-01211-8 (PMC12042486; doi:10.1186/s10020-025-01211-8)

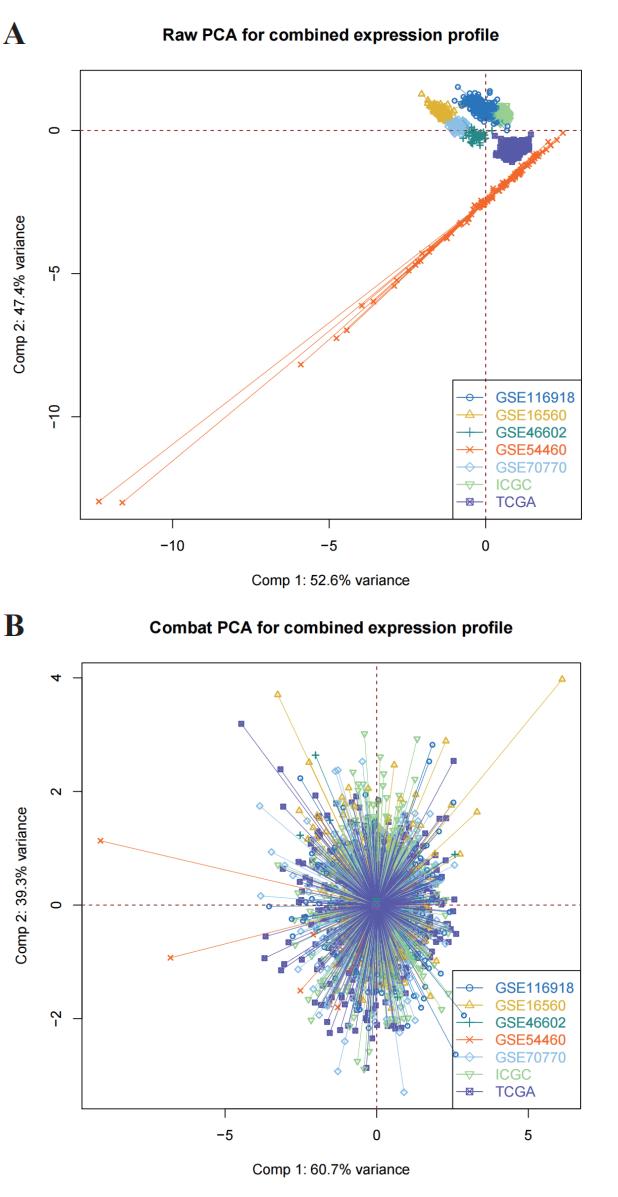

Supplement: Supplementary file 1 — Additional file 1: Figure S1. PCA analysis of gene expression normalization across multiple cohorts. Note:Raw PCA analysis of the combined expression profile across multiple cohorts, showing the initial variance before normalization.PCA analysis after applying the Combat algorithm to normalize gene expression across cohorts, demonstrating that gene expression levels have been successfully corrected to a similar level [file 10020_2025_1211_MOESM1_ESM.jpg]
