# Supplementary material for: TMSB10 drives prostate cancer aggressiveness via immune microenvironment regulation
Source: Mol Med. 2025 Apr 30;31:160. doi: 10.1186/s10020-025-01211-8 (PMC12042486; doi:10.1186/s10020-025-01211-8)
Supplement: Supplementary file 9 — Additional file 9. [file 10020_2025_1211_MOESM9_ESM.docx]

**Immunohistochemistry**

This study compared paraffin samples from patients with benign prostatic hyperplasia/localized prostate cancer and prostate cancer bone metastases, with three samples set per stage (T2/T3/T4).

All paraffin sections were heated at 65°C for 60 minutes, dewaxed twice with xylene (product number: 5340, Sigma-Aldrich), each for 10 minutes, and then sequentially immersed in decreasing concentrations of ethanol (product number: E7023, Sigma-Aldrich) (100%, 100%, 90%, 80%), each for 5 minutes. The sections were then soaked in distilled water for 5 minutes. The slides were then heated in a microwave in sufficient citrate buffer (product number: C9999, Sigma-Aldrich), boiling 4-6 times at 10-minute intervals. After washing 3 times with Tween® 20 (PBST) phosphate-buffered saline, 3% hydrogen peroxide was added, and the sections were sealed at room temperature for 10 min. The slides were washed 3 times with PBS, covered with 10% goat serum, and sealed at 37°C for 60 min. Subsequently, primary antibodies against TMSB10 (product number: bs-1088R, Bioss), PD-L1 (product number: 66248-1-ig; 1:50, Proteintech), IDO1 (product number: sc-53978, Santa Cruz Biotechnology), PD-L2 (product number: sc-80285, Santa Cruz Biotechnology), and ABCAM (product number: ab227679, Abcam) were incubated overnight at 4°C. Secondary antibodies, rabbit (product number: PV‑6000D, Zhongshan Bio‑Tech) and mouse (product number: ab150107, Abcam), Alexa Fluor 594 (TRITC)-affinipure goat anti-rat IgG (H+L) (product number: 112-585-003, Jackson ImmunoResearch), Fluorescein (FITC)- affinipure goat anti-rabbit IgG (H+L) (product number: 111-095-003, Jackson ImmunoResearch) were then incubated at 37°C in the dark for 60 min, followed by 3 PBS soaks.

The treated samples were stained with 3,3'-diaminobenzidine (DAB) (product number: D12384, Sigma-Aldrich) for 1 minute, followed by hematoxylin (product number: H3136, Sigma-Aldrich) for 1 minute, and then with differentiator for 20 seconds. Finally, the samples were dehydrated in ascending concentrations of ethanol (80%, 90%, 1 min, 100%, 100%, each for 2 min) and xylene (twice, each for 2 min), and the sections were sealed with neutral resin for observation.

Researchers blinded to the staging diagnosis evaluated positive areas, randomly selecting three target areas per section. During immunohistochemistry, the mean grayscale value (g) was measured and calculated using the IHC Toolbox plugin of ImageJ (version 1.8.0). A blank control area was also selected, measuring the brightness of the brightest area in the section (g_0_). Optical density (OD) was calculated as follows: OD = log(g_0_/g). Simultaneously, normalized values representing the overall fluorescence intensity of the markers were measured using immunofluorescence. Grayscale values for different samples in each group were statistically described, and variance analysis was used to compare differences in grayscale values between groups.

**Immunofluorescence**

Following the initial sample preparation outlined in the immunohistochemistry section, we conducted TMSB10 immunofluorescence and immune checkpoint analysis on pathological sections. Four slides were selected based on TMSB10 fluorescence intensity (two high TMSB10 and two low TMSB10) for follow-up analysis. The treated sections were incubated with 50-100 µL of 4',6-diamidino-2-phenylindole (DAPI) (product number: 10236276001, Sigma-Aldrich) for 5 minutes at room temperature in the dark, and then fixed with an anti-fade medium. Finally, the samples were dehydrated in ascending ethanol concentrations (80%, 90% for 1 minute each, 100% for 2 minutes twice) and xylene (twice for 2 minutes each), and the slides were sealed with neutral resin for observation.

The processed samples were stained with 3,3'-diaminobenzidine (DAB) (product number: D12384, Sigma-Aldrich) for 1 minute, followed by hematoxylin (product number: H3136, Sigma-Aldrich) for 1 minute, and then differentiated for 20 seconds. The samples were then dehydrated in increasing ethanol concentrations (80%, 90% for 1 minute each, 100% for 2 minutes twice) and xylene (twice for 2 minutes each), and the sections were sealed with neutral resin for observation.

Researchers blind to the staging diagnosis evaluated positive areas, randomly selecting three target regions per section. During immunohistochemistry, the IHC Toolbox plugin of ImageJ (version 1.8.0) was used to measure and calculate the average grayscale value (g). A blank control area was selected to measure the brightness (g_0_) of the brightest area in the section. OD was calculated as follows: OD = log(g_0_/g). Immunofluorescence was used to measure normalized values representing the overall fluorescence intensity of the markers. Grayscale values of different samples in each group were statistically described, and variance analysis was used to compare differences in grayscale values between groups.

**Cell Culture**

We utilized the androgen-dependent prostate cancer cell line LNCaP (product number: CRL-1740, ATCC) and the androgen-independent cell line DU145 (product number: HTB-81, ATCC). HEK-293T cells (product number: iCell-h237, iCell Bioscience Inc.) were used for lentiviral packaging. All three cell lines were cultured in RPMI-1640 medium (product number: 30-2001, ATCC) supplemented with 10% fetal bovine serum (product number: 16140089, Gibco) and 1% penicillin-streptomycin (product number: 30-2300, ATCC) at 37°C in a 5% CO_2_ incubator.

**Transient Transfection**

One day before transfection, cells were seeded in culture plates to reach 70-80% confluency at the time of transfection. A diluted siRNA/nc-lipofectamine 3000 mixture (product number: L3000015, Invitrogen) (see Table 1 for specific sequences) was added to 750 µL of complete culture medium for cell transfection. After 24 hours, the transfected cells were collected for subsequent experiments.

**Construction of Lentiviral Overexpression Vector and Experimental Grouping**

The pLVX-Puro vector (overexpression vector, product number: 632164, Clontech) was used to construct a lentiviral TMSB10 overexpression vector and a negative control. Lentiviral particles were packaged into HEK-293T cells using a lentiviral packaging kit (product number: A35684CN, Invitrogen), and the viral supernatant was collected after 48 hours with a titer of 1×10^8^ TU/mL. For lentiviral-mediated cell infection, 1×10^5^ cells were seeded in 6-well plates. Upon reaching 60-70% confluency, the cells were transfected with an appropriate amount of packaged lentivirus (MOI = 1, working titer ≈1×10^5^ TU/mL) and 5 µg/mL polybrene (product number: TR-1003, Merck). After 4 hours of transfection, an equal medium volume was added to dilute polybrene. Fresh medium was replaced after 24 hours, and cells were subjected to 1 µg/mL puromycin (product number: A1113803, Thermo Fisher) selection after 48 hours to obtain stable cell lines. Overexpression sequences are detailed in Table 2. RT-qPCR was used to verify the overexpression of TMSB10. For the experiment, LNCaP and DU145 cells were divided into the following groups: LNCaP-Control (LNCaP blank control group), LNCaP-pLVX-NC (LNCaP overexpression control group), and LNCaP-pLVX-TMSB10 (LNCaP TMSB10 overexpression group). Similarly, DU145-Control (DU145 blank control group), DU145-pLVX-NC (DU145 overexpression control group), and DU145-pLVX-TMSB10 (DU145 TMSB10 overexpression group) were established. Additionally, groups for LNCaP-Control (LNCaP blank control group), LNCaP-siRNA#1, and LNCaP-siRNA#2, as well as DU145-Control (DU145 blank control group), DU145-siRNA#1, and DU145-siRNA#2 were created. The efficiency of silencing and overexpression was verified using RT-qPCR, with the detailed procedures outlined in the subsequent RT-qPCR methodology section.

**CCK-8 Assay for Cell Proliferation**

Cell proliferation was assessed using the CCK-8 assay kit (product number: CK04, Dojindo). Logarithmically growing LNCaP and DU145 cells were prepared at a density of 5×10^4^ cells/mL, and 100 µL of cell suspension was added to each well of a 96-well plate. Following the abovementioned steps, cells were transfected with siRNA1/siRNA2/NC, and a control group was established. Cells were cultured in an incubator for 24, 48, 72, and 96 hours. After rapidly discarding the supernatant, 10 µL of CCK-8 solution was added to each well, and the plate was incubated at 37°C for 2 hours. The absorbance values (OD) were measured at 450 nm using a Multiskan FC microplate reader (product number: 51119080, Thermo Fisher Scientific). Proliferation rate (%) = [ (OD of the control group - OD of the experimental group) / OD of the control group ] × 100%. Each group had three replicates, and the experiment was repeated three times.

**RT-qPCR**

Total RNA from all treated cell groups was extracted using Trizol (product number: 16096020, Invitrogen). The concentration and purity of RNA were determined based on absorbance values at 260 nm and 280 nm. RNA concentration was calculated as follows: OD260 (OD at 260 nm) × dilution factor × 0.04 μg/μL (for a cuvette with a 1 cm diameter). An OD260/OD280 ratio between 1.8-2.1 indicated high purity of the extracted RNA. For mRNA detection, reverse transcription was performed using a reverse transcription kit (product number: RR047A, Takara) to obtain cDNA, which was then diluted tenfold. QRT-PCR was conducted using the TaqMan Gene Expression Assays protocol (Applied Biosystems, Foster City, CA) with GAPDH as the internal control. The PCR program was set as follows: 95°C for 10 minutes, followed by 35 cycles of 95°C for 15 seconds, 60°C for 30 seconds, and 72°C for 45 seconds. All qRT-PCR reactions were run in triplicate. Primer designs are shown in Table 3. The fold-change in gene expression between the experimental and control groups was calculated using the 2^-ΔΔCt^ method: ΔΔCT = ΔCt of the experimental group -ΔCt of the control group, where ΔCt = Ct of the target gene -Ct of the internal control gene. Ct is the cycle number at which the fluorescence intensity reaches a set threshold, indicating logarithmic amplification. Each experiment was repeated three times.

**Transwell Cell Migration Assay**

After 24 hours of starvation, cells from the treated groups were seeded into the upper chamber of a 24-well Transwell chamber, with 500 µL of medium containing 20% fetal bovine serum added to the lower chamber. After incubation at 37°C for 24 hours, Transwell inserts were removed, cells on the inner layer of the Transwell membrane were wiped off with a cotton swab, rinsed twice with PBS, fixed with 4% formaldehyde, and washed three times with water. Cells were then stained with 0.1% crystal violet for 30 minutes. Images were captured and counted under a Nikon Eclipse Ci microscope (Nikon, Japan) in five fields (200× magnification). Each specimen was replicated three times, and cells were quantified.

**Flow Cytometry for Cell Apoptosis and Cell Cycle Analysis**

Cell apoptosis was detected using the Annexin V-FITC/PI double staining method: LNCaP and DU145 cells, subjected to different treatments, were collected in 15 mL centrifuge tubes and centrifuged at 800 g. The supernatant was discarded, and the cells were washed twice with PBS. Following the protocol of BD Biosciences' apoptosis detection kit (product number: 556547, BD Bioscience, USA), cells were resuspended in 500 μL of binding buffer, then 5 μL of FITC and 5 μL of PI were added and mixed well. The mixture was incubated for 15 minutes in the dark, and cell apoptosis was detected using a BD FACSCalibur flow cytometer. Cells positively stained with Annexin V-FITC were identified as apoptotic. This experiment was repeated three times for consistency.

For cell cycle analysis, LNCaP and DU145 cells, treated differently, were fixed in pre-chilled 75% ethanol for at least one hour and then washed once with PBS solution. A mixture of 400 μL of PI staining solution (50 μg/mL, product number: P4170, Sigma-Aldrich) and 100 μL of RNase A (100 μg/mL, RNASEA-RO, Sigma-Aldrich) was added and incubated for 30 minutes at 4°C in the dark. Cell cycle analysis was conducted on a flow cytometer (BD Biosciences, USA) and analyzed using the ModFit software. This procedure was repeated three times to ensure the reliability of the results.

**Western Blot for Apoptosis and Cell Cycle-Related Proteins**

Following the cell function tests described above, Western blot analysis was performed using specific antibodies to detect cell cycle and apoptosis-related proteins, including TMSB10, BAX (product number: 5023S, Cell Signaling Technology), CASP3 (product number: 14220S, Cell Signaling Technology), BCL2 (product number: 4223S, Cell Signaling Technology), BCL-XL (product number: 2764S, Cell Signaling Technology), CHK1 (product number: 37010S, Cell Signaling Technology), CHK2 (product number: 6334S, Cell Signaling Technology), CDK2 (product number: 18048S, Cell Signaling Technology), P21 (product number: 2947S, Cell Signaling Technology). The antibodies were incubated overnight at 4°C, followed by four washes with 1X TBST buffer, each for 8 minutes. The membranes were then incubated with rabbit secondary antibody (product number: 7074P2, Cell Signaling Technology) at room temperature for two hours, followed by four washes with 1X TBST buffer, each for 8 minutes.

Finally, protein bands were visualized using ECL chemiluminescence (product number: 34580, Thermo Fisher Scientific) and quantified using the Bio-Rad gel imaging system and ImageJ software. Statistical analysis involved a t-test to compare differences between groups, and each experiment was repeated three times.

**Cell Co-Culture System**

A cell Transwell co-culture system was employed to assess the impact of LNCaP and DU145 cells on immune cells. Using 0.4 µm Transwell chambers (3412, Corning, USA), LNCaP and DU145 cells were co-cultured with M1-type macrophages and M2-type macrophages. Cell densities were adjusted to 1×10^5^/mL. M1-type macrophages and M2-type macrophages, sourced from peripheral blood-derived macrophages (CP-H264, Procell), were seeded at approximately 0.8×10^6^ cells per well in a 6-well plate. For M1-type macrophages, 20 ng/ml of IFN-γ and 10 pg/ml of LPS were added. For M2-type macrophages, a final concentration of 20 ng/ml IL-4 and IL-13 each was added to 2mL of RPMI-1640 medium supplemented with 10% FBS and 1% double antibiotics. Macrophages were cultured under these conditions for 48 hours to achieve polarization (PMID: 26253167). Then, 1.5 mL of LNCaP or DU145 cells were seeded in the upper chamber, and 1.5 mL of M1-type and M2-type macrophages were in the lower chamber. Co-culturing was conducted 48 hours in RPMI-1640 medium containing 10% FBS before proceeding with related analyses.

**Cell Viability and Lactate Dehydrogenase (LDH) Activity Assay**

In the predetermined immune cell and target tumor cell co-culture system, cell membrane disruption due to immune cell-mediated cytotoxicity was assessed based on LDH activity levels. LDH activity in co-cultures was detected using an LDH activity assay kit (CAT.NO.C0016, Beyotime, Shanghai, China). LDH is released from the cytoplasm into the culture medium when cell integrity is compromised. Therefore, LDH measurement was used to assess cell damage. Absorbance at 490 nm was measured for each well to determine LDH activity.

**Enzyme-Linked Immunosorbent Assay (ELISA)**

The processed co-culture supernatants were centrifuged at 1000×g for 10 minutes at room temperature, and the supernatants were collected. The levels of IFN-γ (97024ES48, Yeasen Biotech, Shanghai, China), TNF-α (HET019, Bogoo Biotech, Shanghai, China), and IL-10 (EH10245S, Vio Biotech, Shanghai, China) in the cell culture medium were determined strictly following the instructions of the respective kits.

**Flow Cytometry for Cell Sorting**

Different immune cells were sorted as follows: CD80-APC (ab27554, Abcam, UK) and CD86-FITC (ab77276, Abcam, UK) were added for analyzing M1-type macrophages, while CD163-APC (ab134416, Abcam, UK) and CD206-FITC (ab270647, Abcam, UK) were used for M2-type macrophages. Antibodies were incubated for 1 hour in the dark, followed by one PBS wash. Cells were resuspended in 0.5 mL PBS and filtered through a nylon mesh before sorting on a flow cytometer (BD Immunocytometry Systems, USA). Purified cells were cultured in RPMI-1640 medium containing 10% FBS.
